# Intracellular coordination of potyviral RNA functions in infection

**DOI:** 10.3389/fpls.2014.00110

**Published:** 2014-03-26

**Authors:** Kristiina Mäkinen, Anders Hafrén

**Affiliations:** ^1^Department of Food and Environmental Sciences, University of HelsinkiHelsinki, Finland; ^2^Department of Plant Biology and Forest Genetics, Swedish University of Agricultural SciencesUppsala, Sweden

**Keywords:** potyviruses, potyviral RNA functions, potyviral translation, potyviral movement, potyviral replication, potyviral RNA degradation, potyviral RNA encapsidation

## Abstract

Establishment of an infection cycle requires mechanisms to allocate the genomes of (+)-stranded RNA viruses in a balanced ratio to translation, replication, encapsidation, and movement, as well as mechanisms to prevent translocation of viral RNA (vRNA) to cellular RNA degradation pathways. The ratio of vRNA allocated to various functions is likely balanced by the availability of regulatory proteins or competition of the interaction sites within regulatory ribonucleoprotein complexes. Due to the transient nature of viral processes and the interdependency between vRNA pathways, it is technically demanding to work out the exact molecular mechanisms underlying vRNA regulation. A substantial number of viral and host proteins have been identified that facilitate the steps that lead to the assembly of a functional potyviral RNA replication complex and their fusion with chloroplasts. Simultaneously with on-going viral replication, part of the replicated potyviral RNA enters movement pathways. Although not much is known about the processes of potyviral RNA release from viral replication complexes, the molecular interactions involved in these processes determine the fate of the replicated vRNA. Some viral and host cell proteins have been described that direct replicated potyviral RNA to translation to enable potyviral gene expression and productive infection. The antiviral defense of the cell causes vRNA degradation by RNA silencing. We hypothesize that also plant pathways involved in mRNA decay may have a role in the coordination of potyviral RNA expression. In this review, we discuss the roles of different potyviral and host proteins in the coordination of various potyviral RNA functions.

## INTRODUCTION

**FIGURE 1:**
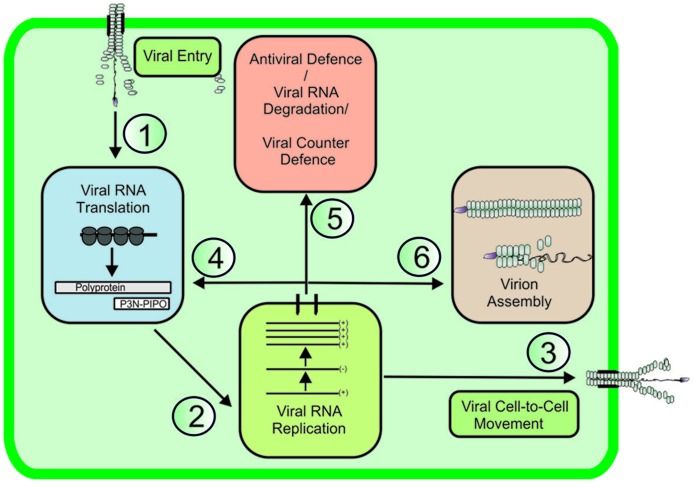
**Viral RNA pathways in infected cell.** In a newly infected cell, polysomes translate viral RNA (vRNA, pathway 1), and it is recruited to VRCs (pathway 2). The replicated vRNA is transported to plasmodesmata to facilitate cell-to-cell movement (pathway 3). To achieve productive infection, vRNA expression continues via new rounds of translation/replication (pathway 4). Host cell defense mechanisms leading to RNA degradation actively compete for vRNA substrates with viral counterdefense mechanisms (pathway 5). vRNA encapsidation completes the infection cycle (pathway 6), allowing the encapsidated virus to be transported and infect neighboring healthy plants.

The replication cycle of positive-stranded (+)RNA viruses involves a chain of several partially overlapping events. The main steps of the replication cycle, namely entry, translation, replication, cell-to-cell movement, antiviral defense/counterdefense, and encapsidation (**Figure 1**), consist of several substeps and a complex regulatory interaction network. In addition to viral proteins, each viral process engages several cellular proteins with either pro- or antiviral functions and it locates to a certain subcellular structure into which the viral RNA (vRNA) and the viral and host proteins involved need to be transported. This review will look at recent developments in understanding the trafficking and functional coordination of potyviral genomes to various pathways in an infected cell (**Figure 1**; pathways 1–6). Potyviruses comprise a very large group of (+)RNA viruses that infect cultivated plants all over the world. Similarly to many other (+)RNA plant viruses, potyvirus infection exploits the protein synthesis machinery of the host in the production of viral proteins; it exploits the endomembrane and cellular secretion systems in the formation of viral replication complexes (VRCs) and plasmodesmata (PD) to enable the spread of the viral genome to other cells (reviewed in Patarroyo et al., 2013).

Although many host factors have already been identified and linked to the RNA synthesis phase within VRCs (Nagy and Pogany, 2011), the molecular details of postreplication events in plant RNA viruses are still mostly sparse. A significant investigative effort is required to elucidate the viral and host proteins involved. While vRNA is multiplying and spreading, it is simultaneously exposed to the virus-induced gene-silencing pathway and likely to some other cellular pathways regulating vRNA decay. Although many functional aspects of gene silencing in antiviral defense and viral counterdefense have been studied in detail (reviewed in Ding and Voinnet, 2007), cell biology studies describing the cellular structures where the antiviral defense and the viral counterdefense take place are lagging behind. Studies of eukaryotic cells have revealed an array of various RNA granules and RNA bodies regulating the host mRNA cycle, metabolism, and gene expression (Anderson and Kedersha, 2006), with various, but still not completely defined, functions in animal virus infections (reviewed in Lloyd, 2013). Similarly, various RNA granules and RNA bodies have been found to exist in plants (Weber et al., 2008; Xu and Chua, 2011; Jouannet et al., 2012), and these may well take part in the regulation of viral (+)RNA functions.

Recently, an interaction network of the *Arabidopsis thaliana*-potyvirus pathosystem, based on experimental reports was proposed (Elena and Rodrigo, 2012). The multiple interactions between viral components and viral and host proteins and between virus targets and their direct partners in this network give an idea of the complexity of the machinery required to coordinate a virus infection. Inevitably, more interactions remain to be found, with an interesting area of study being those required for coordination of potyviral RNA via the formation of ribonucleoprotein (RNP) complexes consisting of host and viral RNA-binding proteins (RBPs). Balanced partitioning of the vRNA substrates to the interdependent pathways competing for vRNA is required to ensure robust and productive infection. Due to the interdependent and sequential nature of the viral processes of (+)RNA virus infection, it is a challenging task to connect the exact step of the viral infection cycle to the correct host and viral RBPs involved.

## FROM vRNA TRANSLATION TO REPLICATION

A prerequisite for replication and the starting point of the infection cycle within a single cell is the translation of viral proteins. The entry of potyviral RNA to initial translation may proceed via two separate routes. First, the virus may be delivered into the host cell from outside (e.g., in the stylet of an aphid). Its subsequent release in the cytoplasm leads to initiation of the replication cycle. Second, the virus may enter a neighboring healthy cell from a previously infected cell, allowing initiation of translation. The establishment of infection within a cell requires a sufficient amount of vRNA to enter the translational machinery without being degraded. Encapsidated potyviral RNA is covalently linked to the viral protein genome-linked (VPg; Oruetxebarria et al., 2001), which may be required for protection of vRNA during the disassembly process prior to or during polysomal translation. Potyviruses employ a genome expression strategy based on the synthesis of a single polyprotein, which is then proteolytically processed to yield 10 individual proteins (reviewed in Rajamäki et al., 2004). In such viruses, the structural and replication proteins are produced in an equimolar ratio. A certain part of the translation events from potyviral RNA leads to production of a shorter polyprotein due to translational +2 ribosomal frameshifting at the 5′terminal part of the P3 encoding gene (Chung et al., 2008; Vijayapalani et al., 2012). This mechanism is used to produce an 11th potyviral protein called P3N-PIPO (pretty interesting potyviral protein, PIPO).

Potyviral (+)RNA serves as a template for both translation and replication. These two functions of vRNA are mutually exclusive, although the exact determinants responsible for the shift from translation to replication have yet to be identified for potyviruses. Interestingly, successful genome amplification and infection require that the translation of the *Tobacco etch virus* (TEV; genus *Potyvirus*) RNA proceeds to a certain position in the coat protein (CP) gene, which is followed by a *cis*-active replication element (Mahajan et al., 1996). The authors suggested that this might provide a mechanism to ensure that only those RNAs that have retained the full open reading frame can be replicated. Recruitment of vRNA to VRCs is likely initiated via interactions of NIb, the RNA-dependent RNA polymerase of potyviruses, and the secondary structures at the 3′UTR of potyviral RNA (Haldeman-Cahill et al., 1998). NIb is recruited to VRCs via its interaction with 6K2-VPg-Pro (Li et al., 1997; Fellers et al., 1998). In addition to NIb and 6K2-VPg-Pro, double-stranded (ds)RNA and likely HC-Pro is localized to the potyviral VRCs, and cylindrical inclusion protein (CI) accumulates as spike-like structures in close proximity (Cotton et al., 2009; Wei et al., 2010; Ala-Poikela et al., 2011). A genome-wide mutagenesis study indicated that most of the potyviral proteins provide essential functions for either genome replication or virion assembly (Kekarainen et al., 2002). Therefore, more viral proteins are likely to exist in VRCs.

(+)RNA viruses, including potyviruses, induce host membrane rearrangements connected to the recruitment of vRNA and replication-associated proteins to assemble VRCs (Miller and Krijnse-Locker, 2008; den Boon and Ahlquist, 2010; Laliberté and Sanfacon, 2010; Verchot, 2011; Grangeon et al., 2012a). The endoplasmic reticulum (ER) membrane is thought to be the site of potyviral translation (Wei et al., 2010). From the ER, vRNA, together with replication proteins, is then captured to initiate replication. The formation of VRCs is initiated by the potyviral membrane spanning-protein 6K2 (Schaad et al., 1997), and ER exit sites (ERES) serve as the platform for the formation of potyviral replication vesicles (Wei and Wang, 2008). Components of the early secretory pathway, namely the Sar1 and Arf proteins, primers of the coat formation for the COP complexes COPII and COPI, respectively, are required for potyvirus propagation (Wei and Wang, 2008). The COPII components Sec23 and Sec24 colocalize with 6K2. As the infection proceeds, the 6K2-containing VRCs fuse with the chloroplast membranes (Wei et al., 2010) with the aid of an ER-derived fusion protein, Syp71 (Wei et al., 2013). The motility of the VRCs is dependent both on the COPII/COPI vesicle trafficking machinery and on the actomyosin system (Wei and Wang, 2008; Wei et al., 2010). Active potyviral replication has been proposed to take place in the chloroplasts, an idea that is supported by the presence of vRNA, dsRNA, and several viral replication proteins in chloroplast-associated VRCs (Wei et al., 2010). *Turnip mosaic virus* (TuMV; genus *Potyvirus*)-induced vesicles have been shown to contain or associate with several host proteins, many of which function in translation, namely eukaryotic initiation factor 4E (eIF4E), eukaryotic elongation factor 1A (eEF1A), RNA helicase-like protein RH8, poly(A) binding-protein (PABP), and heat shock protein 70 (HSP70) (Beauchemin and Laliberte, 2007; Beauchemin et al., 2007; Dufresne et al., 2008; Thivierge et al., 2008; Cotton et al., 2009; Huang et al., 2010). At a later stage of infection, VRCs fused to chloroplasts start to aggregate into tubular structures. Finally, globular structures containing VRCs, chloroplasts, and ER and Golgi markers appear in the perinuclear area of the infected cell (Grangeon et al., 2012b). The latter authors suggested that this structure served as a virus factory, but it is not known how this late-forming structure relates to viral functions (e.g., replication and encapsidation).

The viral protein 6K2 alone is able to direct the formation of ER-derived vesicles, to target them to chloroplasts, and to cause chloroplast aggregation (Wei et al., 2013). As viral replication is not a prerequisite for the cascade leading to the aggregation of 6K2-containing vesicles, it is important to connect the functional replication in space and in time to the correct structures. When visualized with 6K2-GFP, TuMV VRCs were detected at the periphery of chloroplasts by 2 days (Wei et al., 2010, 2013), in VRC-chloroplast aggregations in 50% of infected cells by 3 days and in more than 85% of infected cells by 4 days after initiation of TuMV infection via *Agrobacterium* infiltration (Wei et al., 2013). Although VRC visualization by fluorescent labeling of the 6K2 and subsequent analysis by light microscopy gives a lot of information about the cell biology of potyvirus infection, it doesn’t allow conclusions about the timing of replication. When the timing of *Potato virus A* (PVA; genus *Potyvirus*) replication was tracked by quantitating viral gene expression with *Renilla* luciferase activity and RNA amounts by qRT-PCR a difference in these between wild type and replication-deficient PVA could be detected starting from day 2 after initiation of infection by *Agrobacterium* infiltration. By day 3 the virus had already spread and it had formed infection foci consisting of a substantial number of cells (Eskelin et al., 2010; Suntio and Mäkinen, 2012; Hafrén et al., 2013). These data propose that potyvirus replication is active already at day 2 after *Agrobacterium* infiltration. Although replication and further translation may continue in individually infected cells simultaneously with the movement of vRNA to adjacent cells, many earlier investigations have shown that active potyvirus replication is a transient process (e.g., in *Pea seed-born mosaic virus* infection, PSbMV; genus *Potyvirus*) that takes place in a narrow cell layer at the infection front (Wang and Maule, 1995). A study in protoplasts transfected with *Plum pox virus* (PPV; genus *Potyvirus*) infectious cDNA (icDNA) showed that PPV (-)RNA accumulation reached the maximum at 12 h post-transfection and that RNA amounts decreased to less than 50% of the maximum by 24 h (Raghupathy et al., 2006). Therefore, it is feasible to assume that at a certain point of infection, active replication stops, and vRNA is targeted to postreplication functions.

Current understanding is that the replicated viral (+)RNA is released to the cytoplasm, whereas viral (-)RNA remains in the VRCs (den Boon and Ahlquist, 2010). The spatiotemporal dynamics of the postreplication functions of vRNA in infection are complex. Seminal electron tomography (ET) studies of the *Dengue virus* and *West Nile virus* VRCs revealed high-resolution images of vesicle packages containing viral dsRNA and viral replication-related proteins with pore-like connections. These likely represent the sites from which the replicated RNA is released into the cytoplasm (Welsch et al., 2009; Gillespie et al., 2010). To be able to study the dynamics of the exchange of vRNA and proteins through VRC neck structures, information from high-resolution ET imaging should be combined with sophisticated techniques that allow real time live-cell imaging of vRNA. When such a combination of techniques was exploited to study trafficking of replicated RNA of the *Tick-borne encephalitis virus* from VRCs, released vRNA was found either to associate with ER membranes or to move freely within a defined area of juxtaposed ER cisterna (Miorin et al., 2013). Similar studies need to be carried out with potyviruses to understand how potyviral RNA is released for postreplication tasks. It is not known whether potyviral VRCs contain a neck structure, but it is feasible to assume that progeny (+)RNAs need to be released from VRCs for an infection to proceed. The only potyviral protein suggested to form pores in membranes is the viral genome-linked protein VPg (Rantalainen et al., 2009). This suggestion was based on EM images showing that pore-like structures formed in liposomes containing anionic phospholipids upon interaction with PVA VPg. We propose that PVA VPg could, hypothetically, form a positively charged channel for transportation of vRNA from VRCs, but this area remains to be studied.

## FROM REPLICATION TO CELL-TO-CELL MOVEMENT

Plasmodesmatas are channels that connect the cytoplasm and the ER of two adjacent cells. Plant viruses take advantage of these channels in their cell-to-cell movement, utilizing specialized virus-encoded movement proteins to modify the PD and to target and move vRNA through the PD. Potyviruses encode several proteins, which have a direct role in movement, namely P3N-PIPO, CI, and CP (Dolja et al., 1994, 1995; Carrington et al., 1998; Wei et al., 2010; Wen and Hajimorad, 2010). These proteins, in addition to vRNA, localize to PD (Rodríguez-Cerezo et al., 1997; Roberts et al., 1998) where CI forms conical structures, which are anchored by P3N-PIPO to the PDs (Wei et al., 2010). CI mutants that are not able to support cell-to-cell movement of the potyviral genome cannot reach the PD (Wei et al., 2010). Targeting of CI to the PDs occurs via P3N-PIPO and involves the ER-Golgi secretory pathway. The actomyosin motility system is dispensable for PD localization of these proteins. An interesting feature in the formation of the conical CI structures is their transient nature. These structures were found in PSbMV-infected cotyledons only in the infection stage where active translation and replication take place (Roberts et al., 1998), suggesting that potyviral cell-to-cell movement occurs at an early stage of the infection process. A study following the fate of TuMV CI at different time points of infection reported similar findings (Wei et al., 2010). At a later stage of the infection process, CI aggregated in the cytoplasm into punctate spots. This suggests that at a certain point in the infection process, the cell-to-cell transport machinery is disassembled and cell-to-cell movement ceases.

Potyviral CP has a central role in the cell-to-cell transport of viruses. Assembly deficient TEV cannot support viral cell-to-cell movement (Dolja et al., 1994). The N-terminal domain of CP is important for the assembly of the *Pepper vein banding virus* (PVBV; Anindya and Savithri, 2003) and the cell-to-cell transport efficiency of TEV (Dolja et al., 1994). Phosphorylation of PVA CP regulates both its RNA-binding function (Ivanov et al., 2001) and viral spread in infected plants (Ivanov et al., 2003). These results suggest that the capacity of CP to assemble is an important factor in the cell-to-cell movement of potyviruses. Therefore, the complexes inserted into PDs are likely either assembled virions or viral RNP complexes associated with CP. Both *Potato virus Y* (PVY) and PVA virions are asymmetric and contain a tip structure at the VPg-containing virion end (Torrance et al., 2006). A directional transport function for this structure was proposed and corroborated by the finding that CI, which is an essential cell-to-cell movement factor, associates with this structure (Gabrenaite-Verkhovskaya et al., 2008). In one model, it was proposed that CI associated with the virion tip could serve as the binding site for P3N-PIPO (Vijayapalani et al., 2012). P3N-PIPO is capable of passing through the PD channel and interacting with the host protein PCaP1, a cation-binding protein localized to PD. Vijayapalani et al. (2012) speculated that the potyviral movement complex could be transported through the PD with the aid of the P3N-PIPO-PCaP1 interaction.

An interesting emerging scenario in the cell-to-cell movement of filamentous plant viruses is the close spatial and functional link between replication and movement (reviewed by Tilsner and Oparka, 2012). A recent idea how potyviral RNA could reach PDs is that motile 6K2-containing vesicles enable vRNA transport to PDs. Grangeon et al. (2012a) proposed a model in which the motile vesicles bud at ERES in perinuclear globular structures are trafficked along the ER/microfilaments to the PD. Interestingly, in the next paper from the same authors, it is shown that the motile vesicles derived from the perinuclear globular structures can even pass PD to the adjacent cells (Grangeon et al., 2013). Whether this represents a mode of vRNA transport from cell to cell needs to be studied carefully, but it seems that the globular aggregates form after the conical CI-containing structures have been disassembled from the PDs (Wei et al., 2010). If coreplicational delivery of vRNA to PDs for intercellular movement purposes occurs in potyvirus infection, it likely should occur before the formation of the globular structures in the infection. The role of the intracellular transport machinery in intercellular movement of TuMV was recently demonstrated (Agbeci et al., 2013). Both inhibitors of pre- and post-Golgi transport as well as silencing expression of myosin XI-2 and XI-K genes reduced intercellular TuMV movement (Agbeci et al., 2013) arguing for a role for motile vesicles in TuMV movement. If potyviral RNA is delivered to PDs within VRCs, the question remains as to how CP reaches the PD site and what is the composition of the complex passing through the PD and initiating the infection in the next cell. A possibility is that vRNA is released from motile VRCs at an early stage of the infection process in the vicinity of the PD. A movement complex could then form, with CPs assembling around the vRNA and CI associating with VPg to form the tip. This model shares similarities with another filamentous plant virus group, potexviruses. It was recently shown that *Potato virus X* (genus *Potexvirus*) VRCs gather and dock to the site of PDs (Tilsner et al., 2013). vRNAs released from VRCs in the vicinity of PDs become partially encapsidated by CPs, and they are inserted into the PD channel with the aid of triple gene block 1 protein (TGB1), potexviral RNA helicase. Based on these data Tilsner et al. (2013) proposed a new model of plant viral movement and termed it as coreplicational insertion.

## TARGETING OF REPLICATED vRNA TO NEW ROUNDS OF TRANSLATION/REPLICATION

The number of translation/replication cycles within a single infected cell is an interesting question. The production of progeny viruses may continue until the capacity of the host cell to provide energy, host factors, and host membranes has been fully exhausted by multiple rounds of VRC formation. Tight coupling between potyvirus replication and translation was proposed in two independent studies. Labeling of the TuMV 6K2 protein with two different fluorescent reporter proteins revealed that individual vesicles often carried only one single type of fluorescent label (Cotton et al., 2009). The authors interpreted this as evidence of intimate coupling between a single translated genome and its recruitment to the VRC, and they proposed vesicle-coupled viral translation to explain their observations (Cotton et al., 2009). Another study reported that PVA RNA translation ceases in the presence of a high cytoplasmic concentration of wild-type PVA CP but not in the presence of a mutant CP (CP^mut^), which is deficient in its RNA-binding and particle-formation capacity (Hafrén et al., 2010). In spite of the cytoplasmic excess of wild type CP, PVA RNA encoding for the CP^mut^ was translated. In this case the endogenous CP^mut^ did not affect translation. The authors interpreted that the translation of replicated potyviral RNA likely occurred in an environment not accessible to a cytoplasmic excess of CP and only the endogenous CP could affect translation in this case. Because potyviral CP is able to cease viral gene expression, a mechanism to sequester CP away from the potyviral RNA translation and replication must exist. A study of PVY CP revealed a CP-interacting protein (CPIP) belonging to the family of heat shock protein 40 (HSP40) chaperones (Hofius et al., 2007). Further investigations revealed that CPIP is able to counteract CP-mediated inhibition of PVA gene expression (Hafrén et al., 2010). These authors proposed a model where the delivery of CP via CPIP to HSP70 is utilized to sequester CP from vRNA to allow the vRNA to be translated and replicated until it is time to cease these functions.

Relatively little is known about the molecular determinants and dynamics of the coordination of replicated potyviral (+)RNA to new rounds of protein synthesis/replication. Similar to host mRNAs, vRNAs can be assumed to be associated with RBPs, which are required to protect the integrity of RNA, to suppress RNA degradation pathways, and to coordinate vRNA functions. Assuming that potyviral RNA is transported from VRCs in a similar manner to other (+)RNAs, one possible site for attachment of host proteins to viral RNP complexes is the moment when vRNA or its 5′end enters the cytoplasm. Interestingly, several host proteins that function in translation and/or mRNA regulation associate with (+)RNA VRCs. In the case of potyviruses, these include eIF(iso)4E, PABP, eEF1A, and RH8 (Beauchemin and Laliberte, 2007; Beauchemin et al., 2007; Thivierge et al., 2008; Huang et al., 2010). A limitation in confocal microscopy is that it is not possible to identify the nature of the proteins associated with VRCs (e.g., integral components of the replication machinery or outer surface proteins waiting to target newly synthesized vRNA and transport it to its destination). One possibility is that the role of some of the proteins known to associate with VRCs is to regulate postreplication functions of vRNA, including replication-coupled translation (Cotton et al., 2009; Hafrén et al., 2010).

The determinants of efficient targeting of potyviral RNA in translation have long been discussed. At the heart of these discussions is the VPg -eIF4E/eIF(iso)4E interaction, which was discovered more than 15 years ago (Wittmann et al., 1997). This interaction is required for infectivity (Léonard et al., 2000), its absence is a source of recessive potyvirus resistance (reviewed in Wang and Krishnaswamy, 2012) and potyviral translation is among a number of possible roles that have been linked to it. The eIF4E–VPg interaction increases the affinity of eIF4E to eIF4G (Michon et al., 2006). This may be beneficial for the assembly of the translation preinitiation complex. However, the translation of TEV RNA via a cap-independent mechanism is not dependent on eIF4E (Gallie, 2001) but requires a 5′proximal pseudoknot structure on TEV RNA (Zeenko and Gallie, 2005) and eIF4G rather than eIF(iso)4G (Gallie, 2001; Ray et al., 2006). The interaction of VPg with the poly(A)-binding protein 2 (Léonard et al., 2004) may be required to circularize vRNA for efficient translation. It has been proposed that the VPg molecule serves as a primer for the replication reaction catalyzed by the RNA polymerase NIb (Puustinen and Mäkinen, 2004). This suggestion is based on the finding that potyviral NIb is able to uridylylate VPg in a template-independent manner (Puustinen and Mäkinen, 2004; Anindya et al., 2005). Therefore, the assumption is that the 5′end of the replicated RNA is covalently linked to VPg. This idea was further supported by the finding that encapsidated potyviral RNA contains VPg (Oruetxebarria et al., 2001). It is logical to think that the genome-linked VPg serves in translation functions. However, as discussed below, it is not clear whether this is the case.

**FIGURE 2:**
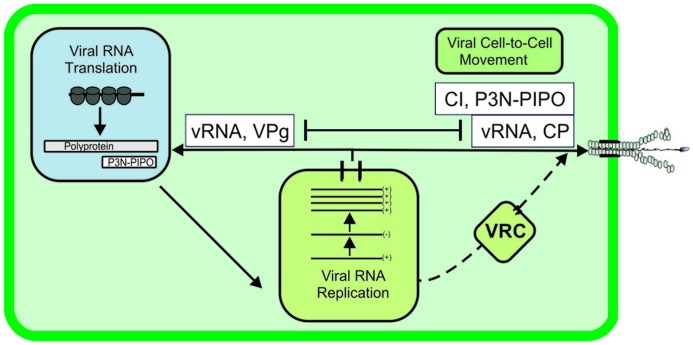
**A model of interdependent targeting of potyviral RNA in translation and movement.** This model of interdependency between vRNA targeting either in translation or movement is based on the observation that VPg, when abundantly present in infected cells, targets vRNA in translation, which is accompanied by a reduction in viral cell-to-cell movement (Hafrén et al., 2013).

Potyviral VPg appears to have a dual role in translation. Slight up-regulation of *in vitro* and *in vivo* translation has been observed in the presences of ectopically expressed VPg with monocistronic template RNAs containing potyviral 5′UTRs, whereas those containing non-homologous UTRs lead to inhibition of translation (Khan et al., 2008; Eskelin et al., 2011). The inhibition of translation by *in trans* given VPg could be explained by VPg-mediated sequestration of eIF4E, reducing its availability for cellular functions. Interestingly, quantitation of PVA gene expression and RNA accumulation in a full infection model revealed that *in trans* given VPg boosts both of these in a concentration-dependent manner (Eskelin et al., 2011). The translation of 5′UTR-lacking vRNA cannot respond to VPg, showing that viral 5′UTR has a central role in VPg-enhanced translation. However, the features of the PVA 5′UTR are not sufficient to explain these observations. The gene expression response of VPg to a construct with PVA 5′UTR in front of a reporter gene was found to be diminutive when compared to that of full-length PVA RNA (Eskelin et al., 2011). Therefore, another still unidentified component of viral origin, either an RNA sequence element or a viral protein, must be required. VPg linked *in cis* to vRNA is not a requirement to achieve VPg-mediated enhanced translation because enhanced translation was detected with non-replicating PVA RNA. The relative level of enhancement was even higher for non-replicating RNA (Eskelin et al., 2011). This is to be expected, given that excess VPg inhibits PVA movement (Hafrén et al., 2013). We propose a tug-of-war model between translation/replication and movement that ensures the correct partitioning of PVA RNA among these two pathways (**Figure 2**). Increasing levels of VPg pull vRNA to translation, leading to concomitant down-regulation of cell-to-cell movement.

The host factors eIF4E/eIF(iso)4E and P0, a ribosomal protein, are involved in VPg-mediated up-regulation of PVA gene expression (Eskelin et al., 2011; Hafrén et al., 2013). The acidic ribosomal protein P0 was identified as a component of the PVA membrane-bound RNP complex. A study of its role in PVA infection revealed a link between VPg-mediated enhancement of PVA RNA translation and P0 (Hafrén et al., 2013). Silencing of P0 led to a significant defect in viral protein and RNA accumulation, whereas there was no delay in viral movement (Hafrén et al., 2013), indicating that this host factor is essential in achieving robust and productive infection. The normal cellular function of P0 is that it is a component of the stalk structure associated with the 60S subunit of ribosomes (Gonzalo and Reboud, 2003), and it plays an essential role in translation. In a study by Hafrén et al. (2013), the other stalk proteins, P1–P3, found in plants (Bailey-Serres et al., 1997; Szick et al., 1998), were not present in a purified PVA RNP complex and did not contribute to VPg-mediated enhancement of PVA translation. In addition, similar to P0 silencing, eIF4E/(iso)4E silencing significantly reduced both vRNA accumulation and viral protein accumulation (Hafrén et al., 2013). In two other studies, silencing of eIF4E led to potyvirus resistance (Mazier et al., 2011; Wang et al., 2013).

The mechanism of recessive resistance to potyviruses caused by incompatibility between eIF4E and eIF(iso)4E and potyviral VPg proteins has long been a puzzle. The resistance mechanism may be dependent on successful formation of an essential RNP complex because many potyviral proteins have the potential to overcome resistance conferred by translation initiation factors in various plants. In addition to the potyviral VPg (Moury et al., 2004; Kang et al., 2005; Ayme et al., 2006; Charron et al., 2008; Gallois et al., 2010), amino acid changes in P1 (Nakahara et al., 2010), P3 (Hjulsager et al., 2006), and CI (Abdul-Razzak et al., 2009) can combat the resistance. *In vitro* interactions between the *Lettuce mosaic virus* (LMV) VPg and helper-component- proteinase (HC-Pro), as well as LMV VPg and eIF4E of lettuce, have been identified (Roudet-Tavert et al., 2007). Research also confirmed the need for eIF4G in LMV infection (Nicaise et al., 2007) and interactions between the C-terminus of CI with VPg and eIF4E (Tavert-Roudet et al., 2012). The interaction network involving VPg, CI, eIF4E, and, possibly, HC-Pro and the translation initiation factor eIF4G were suggested to contribute to the resistance mechanism (Abdul-Razzak et al., 2009). Interactions between potyviral HC-Pro and the initiation factors eIF4E and eIF(iso)4E were also demonstrated (Ala-Poikela et al., 2011). Among the proposed roles for eIF4E/(iso)4E in potyvirus infection are (i) recruitment of the translation initiation apparatus for vRNA translation, (ii) PD targeting of vRNA via VPg, eIF4E, CI, and eIF4G, and (iii) safeguarding virus translation/replication in the cytoplasm via an eIF4E, P1, VPg, and HC-Pro silencing suppressor complex (as reviewed in Wang and Krishnaswamy, 2012). Other possible mechanisms connected to the idea of safeguarding virus translation/replication are the role of VPg–eIF4E interaction in inhibition of cellular mRNA translation to the benefit of the virus and the targeting of vRNA to the virus-specific translational pathway via VPg, P0, eIF4E/(iso)4E and possibly other yet unidentified factors. Due to the central role of VPg, HC-Pro, CI, and eIF4E/(iso)4E in orchestrating the various functions of vRNA, it is feasible to think that a lack of correct interactions between translation initiation factors and these viral proteins may block vRNA access to many interdependent vRNA pathways.

**FIGURE 3:**
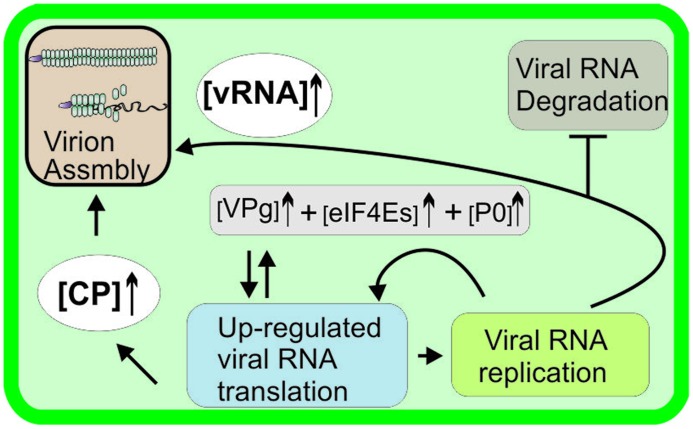
**A model of a virus-specific mechanism to boost viral RNA translation.** P0 transcription is induced in PVY and PVA infection (Baebler et al., 2009; Vuorinen et al., 2010 ) and TuMV infection upregulates eIF4E (Léonard et al., 2004), suggesting that the demand for these proteins is increased during natural potyvirus infection. VPg, eIF4E/(iso)4E, and P0 enhance PVA RNA expression (Hafrén et al., 2013), resulting in the production of a large amount of viral proteins, e.g., CP required for virion assembly at later stages of infection.

Viral gene expression involves a delicate balance. Viral proteins need to be produced in a certain ratio to each other, and even slight alterations may lead to loss of infectivity. Non-structural replication proteins are often required in low amounts, whereas structural proteins need to be produced in massive amounts. As potyviruses employ a genome expression strategy based on polyprotein production, it is not immediately obvious how the regulation of viral protein production is achieved. As suggested by Ivanov and Mäkinen (2012), one possibility is that the VPg-mediated translation pathway boosts vRNA translation in later stages of infection, thereby resulting in the production of a large amount of CP required for virion assembly (**Figure 3**). Such a suggestion is feasible, as conditions to support VPg-mediated translation pathway develop during infection. The more vRNA is translated: the more potyviral VPg is available. Some is transported to the nucleus in the form of NIa (Carrington et al., 1991; Rajamäki and Valkonen, 2009), and some is retained in the cytoplasm for its cytoplasmic functions. PVA infection induces upregulation of P0 transcription (Vuorinen et al., 2010) and TuMV infection eIF4E expression (Léonard et al., 2004), and these proteins may therefore become abundant towards the end of the infection process. The mechanism underlying the cessation of potyvirus gene expression and the shift toward encapsidation needs further investigations.

## STABILITY OF vRNA

The RNA silencing pathway has been shown to compete for potyviral RNA during infection (Kasschau and Carrington, 2001). RNA silencing is a defense mechanism against viruses (Ding and Voinnet, 2007). The silencing pathway consists of initiation, amplification, and systemic spread phases. It is triggered by recognition of vRNA and results in the production of sequence-specific siRNA molecules and siRNA-mediated degradation of vRNA. To counteract RNA silencing, viruses encode for viral suppressors of RNA silencing (VSRs; reviewed in Voinnet, 2005). P1/HC-Pro, the VSR of potyviruses (Anandalakshmi et al., 1998; Brigneti et al., 1998; Kasschau and Carrington, 1998), suppresses RNA silencing either by binding siRNAs (Lakatos et al., 2006) or interfering with siRNA methylation (Ebhardt et al., 2005). Under circumstances of compromised silencing suppression, the amount of TEV RNA is reduced to 17–26% and viral protein accumulation to 10–14% of that found in wild-type infection (Kasschau and Carrington, 2001), showing that potyviral RNA has a strong tendency to be directed to silencing pathways when no protection by HC-Pro is provided. Interestingly, PPV HC-Pro can be replaced functionally by some, but not all, unrelated VSRs (Maliogka et al., 2012), suggesting that interchangeable VSRs share a suppression strategy that allows potyviral RNA to escape the RNA-silencing machinery. It is difficult to link siRNA production patterns directly to their *bona fide* antiviral activities in the presence of VSRs. The essential host factors responsible for RNA silencing in the regulation of potyvirus infection were revealed in a study where a loss-of-virulence phenotype caused by a silencing suppression-deficient HC-Pro mutant was rescued in an RNA-silencing deficient background (Garcia-Ruiz et al., 2010). The dicer-like protein DCL4 and RNA-dependent RNA polymerase 1 (RDR1) and, to a lesser extent, DCL2 and RDR6 were found to be responsible for siRNA production and the antiviral response against TuMV infection in *Arabidopsis*. In agreement with this finding, in another study, down-regulation of RDR1 in transgenic tobacco led to enhanced PVY susceptibility (Rakhshandehroo et al., 2009). Similarly, an essential role for the slicer enzyme Argonaute 2 (AGO2) in antiviral defense against TuMV was demonstrated with the aid of HC-Pro–deficient TuMV in *ago*2 mutant *Arabidopsis* plants (Carbonell et al., 2012).

The exact location of viral siRNA processing is unknown. Various types of RNA bodies have been detected in the cytoplasm of plant cells. The PTGS-related proteins RDR6 and suppressor of gene silencing 3 (SGS3) aggregate in cytoplasmic bodies referred to as siRNA bodies (Jouannet et al., 2012; Moreno et al., 2013). The RNA silencing protein Argonaute 7 (AGO7) has a role in trans-acting small interfering RNA formation in plants (Jouannet et al., 2012). AGO7 colocalizes with RDR6 and SGS3 to siRNA-bodies. Interestingly, AGO7 also partially overlaps with the signal of membrane bound TEV 6K2 (Jouannet et al., 2012). First, this suggests a membrane-association for AGO7 and siRNA bodies. Second, because RDR6 and SGS3 are both essential for plant defense against virus infections, the close proximity of siRNA bodies and TEV 6K2-containing VRCs, prompted the authors to suggest that siRNA bodies may be a point of convergence between viral replication and host defense mechanisms.

Processing bodies (P-bodies) and stress granules (SGs) represent other types of RNA bodies formed in the cytoplasm of plant cells (Xu et al., 2006; Weber et al., 2008). Numerous mRNA decay enzymes, such as the decapping enzymes DCP1 and DCP2, associate with P-bodies, whereas SGs contain translational preinitiation complex components. Balagopal and Parker (2009) proposed an “mRNA cycle” model in which mRNA targeting between translation, degradation, and storage by RNA granules is tightly coordinated. The amount of translational enhancement via VPg is accompanied by proportional enhancement in PVA RNA accumulation (Hafrén et al., 2013). The translational enhancement is not linked to enhanced progeny RNA production via replication because non-replicating PVA RNA accumulates as well, or even better, as replicating RNA. Rather, this finding may reflect the interplay between the partitioning of vRNA to the translation pathway or to a pathway leading to the degradation of vRNA and could relate to the role of PVA VPg in interfering RNA silencing suggested in Rajamäki and Valkonen (2009). The 20- to 100-fold increase in the expression of non-replicating PVA RNA in the presences of excess VPg is an intriguing example of how extensively post-transcriptional RNA regulation can affect gene expression in plants (Eskelin et al., 2011; Hafrén et al., 2013). An increasing body of evidence from animal virus studies indicates that the various types of RNA granules required to regulate cellular mRNA cycle, metabolism, and gene expression are manipulated by RNA viruses to foster more productive replication rates (Lloyd, 2013). Similar responses during plant virus infection are far less studied. Nevertheless, a link between plant virus infection and plant RNA granules may exist, and this is an area that should be investigated in the future.

In addition to RNA silencing, the ubiquitin/26S proteasome system (UPS) may affect potyviral RNA amounts in infected cells (Dielen et al., 2011; Sahana et al., 2012). UPS has been linked to cellular antiviral defense against many viruses in plants (reviewed in Dielen et al., 2010). The 20S proteasome consists of four stacked ring structures, two outer rings formed of seven α subunits, and two inner rings formed of seven β subunits. The two proteasomal enzyme activities which potentially could affect potyvirus infection are protein degradation by proteases (Reichel and Beachy, 2000) and RNA degradation by RNase activity (Ballut et al., 2003; Gautier-Bert et al., 2003). Accumulation of *Papaya ringspot virus* (PRSV) increased when the 20S proteasomal activity in the host plant, papaya, was inhibited (Sahana et al., 2012), indicating that protesome has a role in PRSV infection. PRSV P1 was demonstrated to be prone for proteasomal degradation, whereas the other PRSV proteins were not (Sahana et al., 2012). Interestingly, PRSV HC-Pro when expressed in *Nicotiana benthamiana* leaves mimicked the action of the proteasome inhibitor MG132 and affected both the total amount of ubiquitinated proteins and the amounts of two selected exogenous RNAs of viral and non-viral origin. HC-Pro mutants unable to bind to the proteasomal subunits but still able to bind siRNAs did not cause these effects. The reduction observed in the accumulation of the two mRNAs in the presence of the PRSV HC-Pro mutant deficient in PAA α1-binding is therefore not likely to be due to compromised silencing suppression but to modulated proteasomal activity. PVY HC-Pro interacts with PAA (α1), PBB (β2), and PBE (β5) subunits (Jin et al., 2007) of the *A. thaliana* 20S proteasome, and LMV HC-Pro interacts with the PAE (α5) subunit (Dielen et al., 2011). The interaction site in PVY HC-Pro was mapped to its N-terminus (Jin et al., 2007). The RNase activity of the α5 subunit degraded LMV RNA in an *in vitro* assay, and it was inhibited by LMV HC-Pro (Ballut et al., 2005).

**FIGURE 4:**
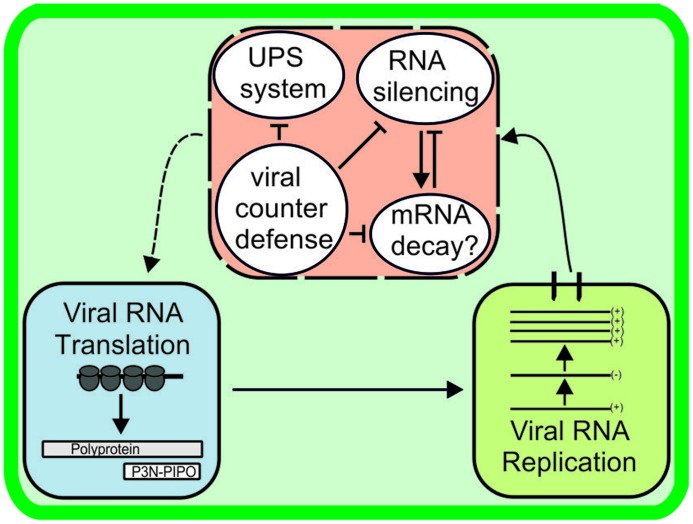
**Viral RNA stability is determined by host antiviral activity and the success of viral counterdefence.** Potyviral RNA may be subjected to degradation by RNA silencing, RNase activity of the ubiquitin-proteasome system, and, as suggested here, mRNA decay pathways unless rescued by viral counterdefense mechanisms. Many links may exist between the RNA degradation pathways. The manner in which vRNA combats viral counterdefense mechanisms and is returned to the active translation/replication pathway (dashed line arrow) is unknown.

An interesting question is how the siRNA-mediated and proteasomal vRNA degradation pathways are related to each other during infection and whether there is a link between these and the other metabolic pathways that regulate the fate of vRNA in cells (Figure 4). Detailed studies on the mechanisms of mRNA decay and RNA silencing in plants have revealed both spatial and functional overlaps (Gazzani et al., 2004; reviewed in Christie et al., 2011). These pathways share RNA substrates, as well as genetic requirements (e.g., mutations in the cytoplasmic exoribonuclease XRN4 and the decapping enzyme DCP2 enhance PTGS; Gy et al., 2007; Thran et al., 2012). Increased transgene silencing phenotype observed in *Arabidopsis* plants that carried a mutation in DCP2 was reverted upon TuMV infection (Thran et al., 2012). The RNA silencing proteins AGO1 and SDE3 colocalized to *Arabidopsis* P-bodies (reviewed in Xu and Chua, 2011). Although not yet linked to virus infection, these examples show the interdependent nature of RNA degradation and storage pathways in plants. With respect to potyviral proteins, VPg and HC-Pro certainly have a role in protecting vRNA against degradation and allowing it to enter the translation/replication pathway rather than the degradation pathway of an infected cell.

## ENCAPSIDATION OF vRNA

Potyviral CP has an essential role in the regulation of infection. In addition to its conventional role in encapsidation, potyviral CP is able to regulate viral gene expression (Hafrén et al., 2010) and movement (Dolja et al., 1994, 1995; Ivanov et al., 2003). In the early phase of virus infection, a mechanism is needed to prevent premature particle assembly and to allow efficient vRNA replication/translation to proceed. Later in the infection, when enough CP has been produced, encapsidation of vRNA is favored. Sequence-specific RNA-protein interactions are often required to initiate the viral assembly process, but the site in the potyvirus RNA where this assembly takes place has not been found. Therefore, the initial CP–RNA interaction site and the mechanism of how potyvirus particles are formed need to be investigated. Potyviruses form capsid shells even in the absence of vRNA, showing that potyvirus particles are largely stabilized by CP–CP interactions. An *in vitro* assembly model was determined for PVBV (Anindya and Savithri, 2003) in which the N- and C-termini of CP subunits interact with each other to form ring-like intermediates, which then assemble to flexous virus-like particles. The sequence of virion assembly *in vivo* may also require the formation of CP intermediates, but this has not been proven yet. CP phosphorylation may provide a mechanism to enable the assembly of potyviruses at the correct time (Ivanov et al., 2001, 2003). Two host chaperones, HSP70 and CPIP (Hofius et al., 2007; Hafrén et al., 2010) were proposed to have a role in controlling CP during active translation/replication (Hafrén et al., 2010). In the proposed model, when the amount of CP produced exceeded the capacity of HSP70/HSP40 chaperones to sequester CP from vRNA, viral encapsidation took place (Nagy et al., 2011). The inhibition of gene expression could be due to the viral assembly process because virion-encapsidated vRNA is not available for translation. However, encapsidation is not the only possible explanation for the inhibition of vRNA expression. Many (+)RNA viruses, such as bromo- and alfamoviruses, regulate their gene expression by specific binding of their CPs to vRNA elements not related to encapsidation (reviewed in Bol, 2005; Kao et al., 2011).

There are many unanswered questions relating to potyviral encapsidation. It is not known where the encapsidation takes place or how the vRNA is transported to the site of encapsidation. The exact mechanism to produce enough CP and to localize it to the site of encapsidation is also unclear. An additional unresolved issue is whether the process of encapsidation requires assistance (e.g., from host chaperones). Previous work suggested that perinuclear globular structures that develop gradually during potyvirus infection provide a site for viral assembly (Grangeon et al., 2012a). This could be plausible given that encapsidation occurs at a late stage in the infection process. However, the presence of virions within these structures still needs to be demonstrated. Another study found that TuMV CP occurs in close proximity to VRCs but does not colocalize with these complexes (Cotton et al., 2009), suggesting that encapsidation could occur at a site adjacent to VRCs. Encapsidated particles contain VPg (Oruetxebarria et al., 2001), which is surface exposed at one end (Puustinen et al., 2002). Thus, VPg is able to participate in protein–protein interactions. A tip structure containing at least CI and HC-Pro attaches to the VPg-containing end of potyviral particles (Torrance et al., 2006; Gabrenaite-Verkhovskaya et al., 2008). The tip structure was observed only in 10% of particles in a purified virus preparation (Torrance et al., 2006), but whether this represents the real ratio of the tip- vs. non-tip-containing viruses in infected cells remains to be investigated. As encapsidation of virions is necessary both for viral movement within the infected plant and transmission of the virus to new hosts by aphids, it is crucial to elucidate the principles underlying virion assembly. Knowledge of particle formation may have practical implications for developing a rational basis for the design of antiviral strategies.

## CONCLUDING REMARKS

Viral RNA trafficking within an infected cell is a tightly coordinated process, which includes many pathways, such as movement, translation/replication, RNA degradation, and encapsidation that compete for the vRNA produced in VRCs. The site of vRNA release from VRCs to the cytoplasm is likely the location of the RNP complexes which allocate functions to vRNA. The varying concentrations of viral and host proteins in different infection phases may be a key factor in determining the fate of vRNA. Coordination of vRNA may be achieved through competition between various RBPs or RNP complexes for the same regulatory elements. Many interesting links have been discovered between the potyviral RNA pathways (e.g., those between replication and movement, as well as vRNA translation and stability). A sophisticated combination of research methods needs to be exploited to dissect the exact roles of various viral and host proteins in these interdependent pathways and to understand the timing and cellular location of each process. Investigations are required to better understand the molecular mechanisms underlying potyvirus infection as a whole and to identify host factors as potential targets for engineering potyvirus-resistant plants.

## Conflict of Interest Statement

The authors declare that the research was conducted in the absence of any commercial or financial relationships that could be construed as a potential conflict of interest.
